# An Easy and Cheap Kiwi-Based Preparation as Vegetable Milk Coagulant: Preliminary Study at the Laboratory Scale

**DOI:** 10.3390/foods11152255

**Published:** 2022-07-28

**Authors:** Fabrizio Domenico Nicosia, Ivana Puglisi, Alessandra Pino, Andrea Baglieri, Rosita La Cava, Cinzia Caggia, Antonio Fernandes de Carvalho, Cinzia Lucia Randazzo

**Affiliations:** 1 Department of Agricultural, Food and Environment, University of Catania, 95123 Catania, Italy; fabrizio.nicosia@phd.unict.it (F.D.N.); ipuglisi@unict.it (I.P.); alessandra.pino@unict.it (A.P.); abaglie@unict.it (A.B.); ccaggia@unict.it (C.C.); 2 ProBioEtna SRL, Spin off of University of Catania, Via Santa Sofia, 100, 95123 Catania, Italy; 3 Caseificio La Cava, 95036 Randazzo, Italy; rosita.lacava@caseificiolacava.com; 4 CERNUT, Interdepartmental Research Centre in Nutraceuticals and Health Products, University of Catania, 14, viale A. Doria 6, 95125 Catania, Italy; 5 InovaLeite—Laboratório de Pesquisa em Leite e Derivados, Departamento de Tecnologia de Alimentos, Universidade Federal de Viçosa, Viçosa 36570 900, MG, Brazil; antoniofernandes@ufv.br

**Keywords:** vegetable coagulant, plant proteases, actinidin, milk-clotting activity, *Actinidia deliciosa*

## Abstract

In the present study, a kiwifruit aqueous extract was developed and used as a coagulant enzyme in cheesemaking. In detail, polyacrylamide gel electrophoresis (SDS-PAGE) was used to investigate the presence of actinidin, the kiwifruit enzyme involved in κ-casein hydrolysis, in different tissues (pulp, peel, and whole fruit) of ripe and unripe kiwifruits. Data revealed the presence of the enzyme both in the peel and in the pulp of the fruit. Although the aqueous extract obtained from the kiwifruit peel was able to hydrolyze semi-skimmed milk, it did not break down κ-casein. The aqueous extract obtained from the pulp showed a hydrolytic activity toward both κ-casein and semi-skimmed milk. The values for milk-clotting and proteolytic activity of the kiwifruit pulp extract were evaluated at different temperatures and pH parameters in order to obtain a high value of the MCA/PA ratio; we found that a temperature of 40 °C in combination with a pH value of 5.5 allowed us to obtain the best performance. In addition, the data revealed a higher hydrolytic activity of the enzymatic preparation from ripe kiwifruits than that from unripe ones, suggesting the use of the extract from pulp of ripe kiwifruits in the laboratory-scale cheesemaking. The data showed that 3% (*v*/*v*) of the ripe kiwifruit pulp extract determined a curd yield of 20.27%, comparable to chymosin yield. In conclusion, the extraction procedure for kiwifruit aqueous extract proposed in the present study was shown to be a fast, cheap, chemical-free, and ecofriendly technology as a plant coagulant for cheese manufacturing.

## 1. Introduction

Milk-clotting proteases are essential enzymes for cheesemaking, and among them, animal rennet, which is extracted from the abomasum of the newborn ruminants, is the most widely used. It contains a high amount of chymosin (EC 3.4.23.4), an aspartic protease that can hydrolyze a specific κ-casein bond (Phe_105_-Met_106_), thus causing the coagulation of milk during cheesemaking [1]. The worldwide increase in cheese production, combined with the reduction in supply and the increasing prices of calf rennet [2], as well as religious components (in Islam and Judaism) and factors related to vegetarianism, have led to the search for alternative enzymes for coagulation of milk as appropriate substitutes for animal rennet [3]. Microbial coagulants are the most commonly substituted enzymes available; they are aspartic proteases produced by *Rhizomucor miehei* and *Rhizomucor pusillus* used to obtain various types of cheeses [4]. These enzymes have a low production cost, but also have some defects such as heat resistance, which involves a high proteolytic activity and therefore defects in the cheese (bitterness, low cheese yield) [5]. The development of recombinant DNA technology allowed the creation of fermentation-produced chymosin (FPC) as an innovative substitute for animal rennet. This low-cost technology clones the bovine chymosin gene into a host microorganism that will produce it through fermentation [6], which obtains chymosin with the same characteristics of the animal one. By using EPS, it is possible to obtain cheese with excellent quality [6]. However, technologies involving recombinant DNA are the subject of great debate for ethical reasons, and many countries, such as France, Germany and the Netherlands, have restricted the use of FPC [7,8]. Nowadays, a completely natural alternative is represented by plant-derived milk-clotting enzymes, which are commercialized and used as valid substitutes for animal rennet [3]. They have become of growing interest in the cheese industry due to their easy availability and simple purification processes. Among them, proteases are present in tissues of various plants, such as cardoon flower [9], *Cynara scolymus* artichoke [10], ginger (*Zingiber officinale*) rhizomes [11], *Dregea sinensis* stems [12], and *Citrus aurantium* flowers [13]. Many of these enzymes have been widely used for the production of the Portuguese and Spanish soft cheese types. However, due the excessive proteolytic activity, plant-derived enzymes generate bitter flavors, limiting their industrial use. Among plant-derived enzymes, kiwifruit extract was revealed to have promising milk-clotting properties. Kiwifruit (*Actinidia deliciosa*) contains high amounts of actinidin (EC. 3.4.22.14), a cysteine protease, which showed a high potential for its use as a milk-clotting agent in cheesemaking [14]. Actinidin is composed of 220 amino acid residues with a molecular mass of approximately 23.5 kDa [15]. The optimal parameters for actinidin activity, such as pH and temperature, are compatible with those used during cheesemaking. Actinidin and hydrolyze β-casein, followed by κ-casein at several points (Arg_97_–His_98_ or Lys_111_–Lys_112_ bonds), possess a higher specificity of hydrolysis against caseins than other proteases (such as papain) [16]. Mazorra-Manzano et al. (2013) [17] demonstrated that cheese made with kiwifruit extract showed a higher dairy yield, chewiness, springiness, and gumminess than cheeses produced with melon (*Cucumis melo*) or ginger rhizomes (*Zingiber officinale*). In addition, kiwifruit extract revealed the highest milk-clotting activity (MCA)/proteolytic activity (PA) ratio, thus representing the most effective alternative to calf rennet. However, the purification of kiwifruit extracts is a complex and time-consuming process, and often requires expensive equipment [14,16,17,18]. 

In the present study, a cheap, quick, and easy kiwifruit aqueous preparation was developed and used as a coagulant enzyme at the laboratory scale. The kiwifruit extract was obtained from both the pulp and peel of the fruit at different ripening times, and different extract concentrations of ripe fruit pulp were tested in a cheesemaking trial at the laboratory scale to establish the effect of the vegetable coagulant on the yield of the cheeses. 

## 2. Materials and Methods

### 2.1. Raw Materials

The kiwifruits (*Actinidia deliciosa* cv. Hayward) used to obtain the aqueous extracts were purchased at a local market in Catania, Italy. The fruits were preliminarily evaluated for their ripeness degree using a digital refractometer (Atago, RX-5000, Tokyo, Japan): unripe (9.5° Brix) and ripe (14° Brix). Cow’s milk was kindly provided by the La Cava dairy farm (Randazzo, Italy), and was used for cheesemaking at the laboratory scale.

### 2.2. Kiwifruit Aqueous Extracts Preparation

Unripe and ripe kiwifruits were washed, peeled with a knife, weighted, and pulped. The aqueous extract of the kiwifruit pulp was obtained by pressure using a manual stainless-steel press. The obtained juice was filtered twice through sterile gauze (50 grade, 20 × 20 cm) to separate all the seeds and the coarse content from the juice. The residual peel was blended (30 s) and then filtered through two layers of sterile gauze to obtain the peel’s aqueous extract. 

Whole fruits were separately weighted, washed, blended (30 s), pressed (as above), and filtered to obtain whole-fruit aqueous extract following the same extraction procedure used for pulp. The kiwifruits were processed to obtain different aqueous extract preparations as follows: (i) ripe fruit pulp, (ii) ripe fruit peel, (iii) ripe whole fruit, (iv) unripe fruit pulp, (v) unripe fruit peel, and (vi) unripe whole fruit.

Aqueous extract yields of kiwifruits were expressed as mL of obtained juice per kg of whole kiwifruits processed. Finally, samples of 10 mL of each extract were placed in an convective oven at 105 ± 2 °C until reaching a constant weight (24 h) to determine the dry matter (DM) of the extracts.

### 2.3. Electrophoretic Analyses

An aliquot of all kiwifruit aqueous extract preparations was centrifuged for 10 min at 5000 rpm at 4 °C; then the supernatant was recovered and used for electrophoretic analyses. The protein content of extract samples was assessed using a Pierce™ BCA Protein Assay Kit (Thermo Fisher Scientific, Waltham, MA, USA). Extract samples (2 µg in protein) were prepared for SDS-PAGE by adding an equal volume of loading buffer (62.5 mM Tris-HCl, pH 6.8, 2% SDS, 25% glycerol, 0.01% bromophenol blue, and 5% 100 mM DTT). 

The hydrolytic action of the aqueous extracts from the different tissues of ripe and unripe fruits toward κ-casein (Sigma, Darmstadt, Germany) and semi-skimmed cow milk was also evaluated. The hydrolysis was performed according to Puglisi et al. (2014) [14]. Partially skimmed milk and κ-casein (10 mg in protein) dissolved in 67 mM NaH_2_PO_4_ pH 7.2 buffer (final volume 300 µL) were used as substrates, and were incubated with each aqueous kiwifruit extracts (10 µg protein) for 20 min at 55 °C. Aliquots of 10 µL samples were loaded into gel wells. 

SDS-PAGE patterns (4–20% slab gels) were determined according to the method of Laemmli (1970) [19]. After the electrophoresis run was over (24 mA for 6 h), the gels were immersed for 8 h in a dye solution (50% methanol, 7.5% acetic acid, and 0.2% Comassie Blue R-250). The excess dye was removed via several washings (for about 4 h) with a bleaching solution (15% methanol and 7.5% acetic acid). 

### 2.4. Milk-Clotting Activity Determination

Based on the results obtained from the electrophoretic analysis, further tests were conducted only on the extract obtained from ripe kiwifruit pulp. The milk-clotting activity (MCA) was determined as described by Arima et al. (1970) [20] with slight modifications. In detail, the aqueous extract obtained from pulp of ripe fruits was stabilized by adding an equal volume (*v*/*v*) of 20 mM sodium phosphate buffer (pH 7.0), then 1 mL of coagulant was added to 10 mL of low-fat (1%) pasteurized milk (containing 0.02% CaCl_2_). The period elapsing between inoculation with the coagulant and the appearance of the first clot was calculated and expressed as the clotting time (seconds). The MCA was defined in terms of the Soxhlet unit (SU), representing the amount of protein in 1 mL of coagulant able to clot 1 mL of low-fat milk in 40 min (2400 s), and was expressed as: MCA (SU) = 2400/t × S/E, where t = clotting time (sec), S = volume of milk (mL), and E = volume of extract (mL).

The MCA was tested at different temperatures (35 °C, 40 °C, 45 °C, 50 °C, and 55 °C) and pH values (5.2, 5.5, 6.0, 6.5, 7.0, and 7.5). The temperatures were settled using a thermostatic bath (WB-M50, Falc Instruments, Treviglio, BG, Italy), and the MCA was assayed at a constant pH of 7.0. The pH effect was monitored at the optimal temperature of 40 °C by adjusting milk samples at the different pH values and monitoring with a digital pH-meter (MettlerDL25, Mettler-Toledo International Inc., Columbus, OH, USA). The assay was performed in triplicate.

### 2.5. Proteolytic Activity Determination

Proteolytic activity (PA) was determined using the method of Kunitz (1947) [21] with low-fat milk powder as the substrate. Briefly, 50 μL of kiwifruit aqueous extract from the pulp of ripe fruits was added to 450 μL of 1% substrate solution (0.1 M phosphate buffer, pH 7.0) and incubated at 40 °C for 60 min. After incubation, the reaction was stopped by the addition of 500 μL of 5% (*w*/*v*) trichloroacetic acid (TCA); for the control sample, the TCA was added immediately before incubation, and then the sample was placed on ice. The mixture was vortexed (ZX3, Velp Scientifica, Usmate Velate, MB, Italy), left to stand on ice for 30 min, and then centrifuged at 13,000 rpm for 20 min. The optical density (OD) of the supernatant was then measured at 280 nm using a Perkin-Elmer Lambda 1 spectrophotometer. One unit of enzyme activity (U) was defined as the amount of protein that increased the absorbance by one unit at 280 nm under the conditions described above. PA was tested at different pH values (5.2, 5.5, 6.0, 6.5, 7.0, and 7.5) and at the optimum temperature of MCA (40 °C) using 0.1 M sodium phosphate buffer at the different pHs. The pH value was monitored using a digital pH meter (MettlerDL25, Mettler-Toledo International Inc., Columbus, OH, USA). The assay was performed in triplicate.

### 2.6. Cheesemaking at the Laboratory Scale

The cheesemaking was carried out at the laboratory scale following the method of Cologna et al. (2009) [22] with slight modifications, by placing Pyrex beakers in a thermostatic bath using 500 mL of cow milk at the optimal MCA temperature (40° ± 1). Once the temperature of 40 °C was reached, lyophilized commercial starter cultures, provided by the La Cava farm, were added for milk acidification until reaching a pH of 5.5 ± 0.2, the optimal pH for the highest MCA/PA ratio. Different amounts of aqueous extract, corresponding to 1.6, 2.0, 3.0, and 4.0% *v*/*v*, obtained from the pulp of ripe kiwifruits were added to the milk samples. When coagulation was completed, the curd was broken into very small irregular granules (4–6 mm) with a thorn, then pressed for 15 min in a cheese mold. The curd was turned and pressed for 30 min to facilitate the purging of the whey. Curd yields were calculated as curd weight/milk weight × 100. Chymosin and microbial coagulant (supplied by Caglificio Clerici, Como, Italy) were used as a control. The cheesemaking at the laboratory scale was conducted in triplicate.

### 2.7. Statistical Analysis

One-way ANOVA analysis with Tukey’s post hoc test was applied in the MCA determination, PA determination, and cheesemaking test in three replicates using Statistica software (TIBCO Software, Palo Alto, CA, USA) to evaluate the statistical differences between the samples. Differences were statistically significant at *p* < 0.05.

## 3. Results 

### 3.1. Kiwifruit Aqueous Extracts Preparation

The aqueous kiwifruit extract preparation was obtained by using the procedure described in Section 2, and showed extract yields of 364 mL/kg and 457 mL/kg from unripe and ripe kiwifruit pulp, respectively. Similarly, yields of the aqueous extracts obtained from the whole ripe and unripe fruits were 469 mL/kg and 371 mL/kg, respectively, as shown in Table 1. Yields from the peels of both ripe and unripe fruits were low (15 mL/kg and 11 mL/kg, respectively). 

### 3.2. SDS-PAGE Electrophoretic Profile

**Figure 1:**
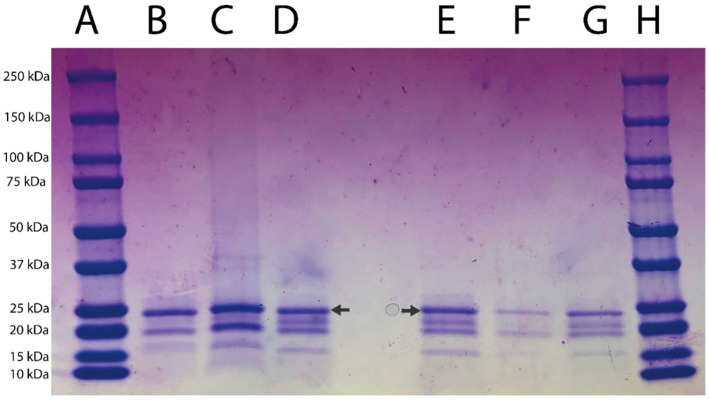
SDS-PAGE patterns of aqueous extract of ripe and unripe kiwifruits. Lanes A, H: molecular markers; lane B: ripe fruit pulp; lane C: ripe fruit peel; lane D: ripe whole fruit; lane E: unripe fruit pulp; lane F: unripe fruit peel; lane G: whole unripe fruit. The arrows indicate actinidin bands corresponding to approximately 23 kDa.

**Figure 2:**
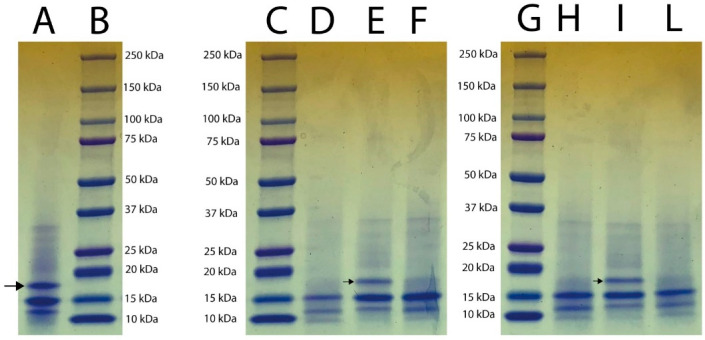
SDS-PAGE patterns of κ-casein subjected to treatment with aqueous extracts of kiwifruit tissues. Lane A: control κ-casein; lanes B, C, G: molecular markers; lane D: ripe fruit pulp; lane E: ripe fruit peel; lane F: ripe whole fruit; lane H: unripe fruit pulp; Lane I: unripe fruit peel; lane L: whole unripe fruit. The arrows indicate the band corresponding to κ-casein (19 kDa, Sigma).

**Figure 3:**
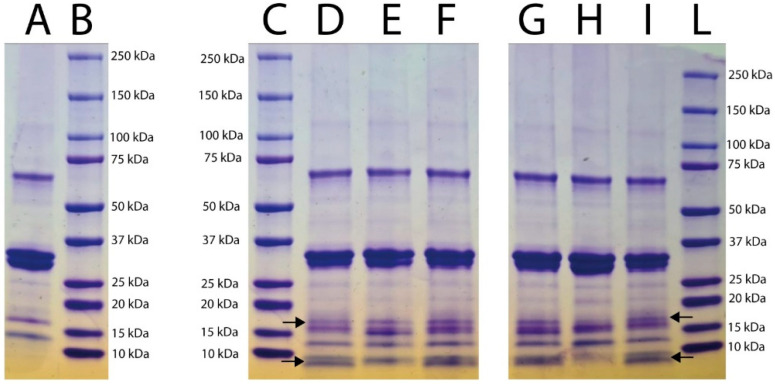
SDS-PAGE patterns of semi-skimmed milk treated with aqueous extract of kiwifruit tissues. Lane A: control semi-skimmed milk; lanes B, C, L: molecular marker; lane D: ripe fruit pulp; lane E: ripe fruit peel; lane F: ripe whole fruit; lane G: unripe fruit pulp; Lane H: unripe fruit peel; lane I: whole unripe fruit. Arrows indicate the peptides formed after milk hydrolysis.

The presence of actinidin in the fruit tissues at different ripeness degrees was determined using electrophoresis; Figure 1 shows the pattern profiles of extracts from ripe fruit pulp (lane B), ripe fruit peel (lane C), ripe whole fruit (lane D), unripe fruit pulp (lane E), unripe peel (lane F), and unripe whole fruit (lane G). The results showed the presence of two main bands of approximately 20 kDa and 23 kDa in all tissue samples. The band of approximately 23 kDa corresponded to that of actinidin’s molecular weight, as previously reported [23]. The hydrolysis electrophoretic patterns of κ-casein subjected to the enzymatic action of kiwifruit aqueous extracts are reported in Figure 2. The band with a molecular weight of 19 kDa, corresponding to κ-casein as reported by the producer (Sigma), was completely hydrolyzed by the aqueous extracts from the pulp of ripe fruits (Figure 2, lane D). Similarly, extracts from the pulp of unripe fruits (Figure 2, lane H) hydrolyzed κ-casein, but to a lesser extent than the extract from pulp of ripe ones. On the contrary, the aqueous extract from the peels of both ripe and unripe kiwifruits (Figure 2, lanes E and I) did not hydrolyze κ-casein, which remained intact after treatment, as indicated in Figure 2 (lanes E and I) by arrows. Finally, a partial hydrolysis of κ-casein due to the action of extracts from both ripe and unripe whole fruits was detected (Figure 2, lanes F and L). Figure 3 shows the patterns of semi-skimmed milk treated with the kiwifruit aqueous preparation. In all samples, hydrolysis of the milk proteins produced peptides showing an apparent molecular weight ranging from 8 to 9.6 kDa. These bands were less evident in milk treated with extracts from peel samples (Figure 3, lanes E and H), suggesting that a different degree of hydrolysis of the milk occurred. Furthermore, a band with a molecular weight of 16.9 kDa was clearly detected in all samples.

### 3.3. Temperature and pH Effects on Milk-Clotting Activity (MCA)

**Figure 4:**
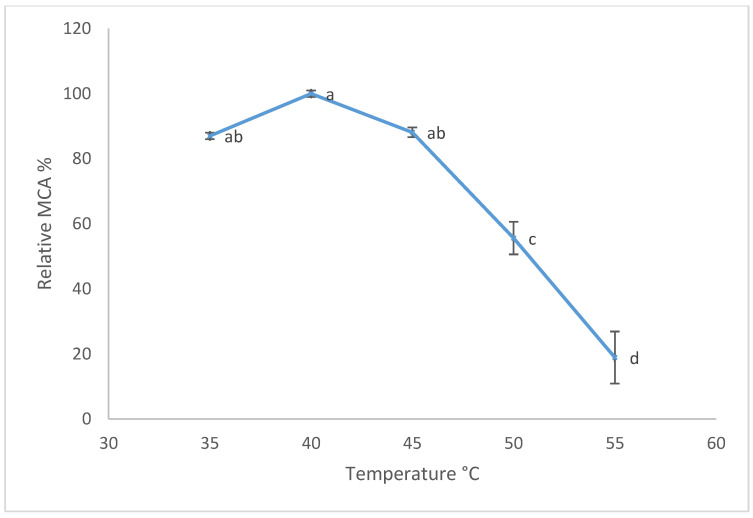
Temperature’s effect on MCA of aqueous extract of pulp ripe kiwifruits. The percentage (%) of relative MCA represents the mean of three independent determinations performed in triplicate. The maximum value of MCA was 100%. Error bars represent standard deviations. Different lowercase letters (a, b, c and d) indicate a significant difference among samples at *p* < 0.05 (ANOVA with Tukey’s post hoc test).

**Figure 5:**
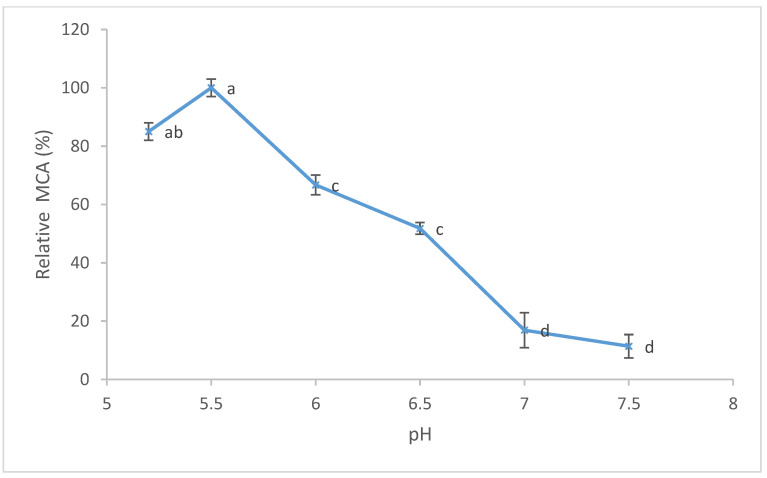
Effect of different pHs on milk-clotting activity of aqueous extract from ripe pulp kiwifruits. The percentage (%) of relative MCA represents the mean of three independent determinations performed in triplicate. The maximum value of MCA was 100%. Error bars represent standard deviations. Different lowercase letters (a, b, c and d) indicate a significant difference among samples at *p* < 0.05 (ANOVA with Tukey’s post hoc test).

Based on previous results, the aqueous extract from the pulp of ripe kiwifruits was used for further experiments. The effect of temperature on MCA is shown in Figure 4. The maximum (100%) MCA value (3.87 SU/mL) was reported at a temperature of 40 °C. The MCA slowly decreased when the temperature reached 45 °C (3.41 SU/mL), showing a drastic decrease at 55 °C (0.73 SU/mL). Furthermore, the effect of pH (ranging from 5.2 to 7.5) on MCA was evaluated, and the results are shown in Figure 5. The maximum (100%) value of MCA was detected at pH 5.5 (5.43 SU/mL), which decreased at pH 6.0 and above.

### 3.4. Effects of Different pHs on Proteolytic Activity (PA) and MCA/PA Ratio

**Figure 6:**
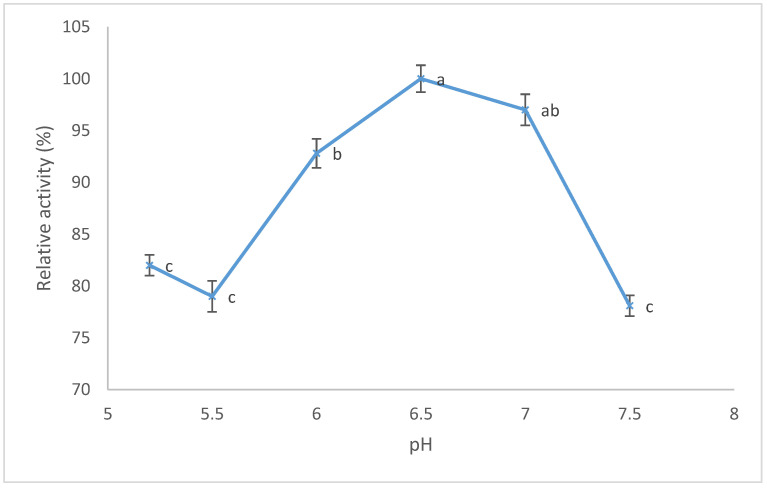
Effect of different pHs on proteolytic activity (PA) of aqueous extract from ripe pulp kiwifruit. The percentage (%) of relative PA represents the mean of three independent determinations performed in triplicate. The maximum value of PA was 100%. Error bars represent standard deviations. Different lowercase letters (a, b and c) indicate a significant difference among samples at *p* < 0.05 (ANOVA with Tukey’s post hoc test).

The proteolytic activity was tested at different pH values (from 5.2 to 7.5) at a constant temperature of 40 °C (which was the best temperature previously determined for MCA), and results are shown in Figure 6. Proteolytic activity (PA) showed the maximum value (0.754 U/mL) at pH 6.5, then a slight decrease at a more acid or alkaline pH. At pH 5.5 (the best pH for MCA), a lower PA value was recorded (0.596 U/mL), suggesting that this pH may be a suitable value for an optimal condition for cheesemaking. Moreover, Table 2 shows the MCA/PA ratio at different pH values.

### 3.5. Cheesemaking Test

A cheesemaking trial at the laboratory scale was conducted using different amounts of the aqueous extract from the pulp of ripe kiwifruits. Table 3 reports the results for yields (%) of curds obtained from different percentages of the extract. In addition, the data were compared to those obtained from chymosin and microbial coagulant. The data revealed that a percentage (20%) of 3 (*v*/*v*) and 4 (*v*/*v*) of the kiwifruit extracts showed a comparable yield to those of chymosin and microbial coagulants.

## 4. Discussion

Plant-derived enzymes have become of growing interest in dairy technology. Among them, actinidin, a cysteine protease from kiwifruit, is a promising substitute for chymosin due to its ability to form a good milk clot; moreover, the enzyme is fully compatible with technological parameters used during cheese manufacturing. Currently, several kiwifruit-extract preparation methods are already available in the literature [14,16,17,18,24]; however, they are time-consuming and high-cost procedures. 

In the present study, a fast and cheap preparation of the aqueous extract of kiwifruit was developed and used for laboratory-scale cheesemaking. A higher yield in juice extract may be obtained by using ripe kiwifruits as a starting material, thus allowing the use of waste fruits with a high ripeness degree that are discarded by the food industry in the preparation of different kiwi-based foods and drinks. The data revealed that the kiwifruit aqueous extract from ripe fruit showed a higher clotting yield than that of unripe ones, suggesting a higher actinidin concentration in ripe fruit. It was noteworthy that the concentration of protein in kiwifruit is related to the growth stage, cultivar types, and treatment of the fruit during postharvest storage [25,26]. Our data from the SDS-PAGE profile suggested that the quantity of actinidin in the fruits’ tissues was strongly dependent on the ripeness degree. These data were supported by other studies that demonstrated that plant coagulants, such as those prepared using Noni (*Morinda citrifolia* L.) fruits [27] and berries of *S. elaeagnifolium* [28], were obtained from ripe fruits. The hypothesis that the ripeness degree of the fruit positively influenced the presence and the activity of the actinidin enzyme was also confirmed by Karki et al. (2018) [29], who found that the amount of actinidin was greater in ripe kiwifruits, which showed a higher level of activity (299 U/mg) against total casein with respect to unripe fruits. 

In the present study, the presence of actinidin in the peel samples was also revealed, confirming the results of the study conducted by Nieuwenhuzen et al. (2007) [30], in which the amount of actinidin was determined both in the pulp and in the peel of the fruits. However, although actinidin was detected in the peel samples, the hydrolysis electrophoretic pattern of the kiwifruit aqueous extracts toward κ-casein showed that extracts obtained from the peel of the fruits, both ripe and unripe, seemed unable to hydrolyze κ-casein (corresponding to the band at 19 kDa). The factors affecting hydrolysis activity toward κ-casein could be the presence of actinidin in the peel of the fruit, which was in a minimal quantity compared to the content present in the pulp, as reported by Lewis and Luh (1988) [31]; or an insufficient extraction of the target components from the peel during the procedure. Moreover, a lower hydrolysis pattern on κ-casein treated with the extract of whole kiwifruits was shown with respect to that obtained using pulp extract. This could be explained by the possible presence of substances in the peel of kiwifruits, which can determine a putative inhibition of κ-casein hydrolysis. The patterns of semi-skimmed milk treated with the aqueous extract of kiwifruits showed, in all the samples, bands of about 8–9.6 kDa, which were similar to those generated by the hydrolysis of semi-skimmed milk after the treatment with pure actinidin (Lo Piero et al., 2011) [16]. Moreover, according to Chalabi et al. (2014) [32], bands corresponding to 16.9 kDa may be the result of hydrolysis produced by actinidin upon α-casein. These results were also comparable to those obtained by Puglisi et al. (2014) [14], which showed a digestion product with a molecular weight of 16.9 kDa generated by the hydrolytic action of an aqueous kiwifruit extract toward semi-skimmed milk. The aqueous extract from the pulp of ripe fruits showed an optimum temperature for MCA at 40 °C, in agreement with Mazorra-Manzano et al. (2013) [17]. This optimum temperature revealed that this preparation may be compatible with industrial cheesemaking. The MCA slowly decreased when the temperature reached 45 °C and 55 °C, probably due to the protease denaturation after the heat treatment. This result was comparable with that reported by Lo Piero et al. (2011) [16], who demonstrated that when 55 °C was reached, the caseinolytic activity of actinidin was reduced by 30%. Finally, the maximum value of MCA detected at pH 5.5 was in agreement with results obtained by Grozdanovic et al. (2013) [23] and Chalabi et al. (2014) [32], who demonstrated that actinidin showed better performances at acid pHs ranging between 5.0 and 5.5.

The proteolytic activity (PA) of a clotting enzyme is an important parameter for cheese ripening; high values of proteolysis are often associated with the formation of off flavor and bitter taste due to the production of short peptides [7,23]. The maximum value of proteolytic activity of the aqueous extract from the pulp of kiwifruits was reached at pH 6.5, and decreased at more acidic or alkaline pHs (Figure 6), in agreement with Lo Piero et al. (2011) [16]. Dehkordi et al. (2021) [33] found that, although kiwifruit extract was a good substitute for animal rennet during cheesemaking, the use of this extract could lead to defects in the cheese (bitterness and soft texture) caused by its high proteolytic activity. Katsaros et al. (2009) [34] inactivated the actinidin after coagulation of the cheese using high hydrostatic pressure (HHP) to reduce the defects caused by the high proteolytic activity of the kiwifruit extract. Another method that allows researchers to obtain a better cheese is to vary the parameters of pH and temperature in order to manage the MCA/PA ratio, as high values of the MCA/PA ratio correspond to a coagulant capable of providing a cheese free of defects such as bitter flavors [35]. In the present work, the highest calculated MCA/PA ratio was 9.1, and it was reached at 40 °C and a pH of 5.5. The aqueous extract at pH 6.0 reached an MCA/PA ratio of 5.2, which was similar to the ratio of 5.0 calculated by Mazorra-Manzano (2013) [17]. 

The search for the suitable amount of coagulant is a critical point in cheesemaking, as an excess of proteases can influence secondary proteolysis (developing defects such as bitter flavors), while insufficient quantities lead to a loss in consistency of the cheese [5]. Moreover, a long exposition to protease action can determine a proteolytic degradation of the casein network (especially α- and β-casein), thus reducing approximately 0.3–0.7% the curd yield [36]. In this work, curd made with an inoculum of 3% (*v*/*v*) of aqueous extract from pulp of ripe fruits provided a yield of 20.27%, and similar results were achieved when 4% of the coagulant was used (20.03%). These results suggested that an inoculum of about 3% (*v*/*v*) is enough to obtain a maximum curd yield that is comparable to the yields obtained using chymosin (20.93%) and microbial coagulant (20.06%). Lower yield values were found in similar studies. Mazzorra-Manzano et al. (2013) [17] used an extract of kiwifruit prepared from slices of peeled fruit stabilized by adding one equal part (*w*/*v*) of 20 mM sodium phosphate buffer (pH 7.2) and then homogenizing in a blender, obtaining a yield of 17.8% coagulated bovine low-fat milk. Ojha et al. (2021) [37] reached a yield of 16.69% (goat milk) using a kiwifruit extract obtained after pressing the pulp and centrifuging it at 3000 rpm for 10 min as a coagulant. A statistical analysis showed that only the samples inoculated with 2% and 1.6% of the extract produced significantly different results, which meant that both amounts were not enough to reach an optimal yield.

The curd yield produced using 3% of the extract from the pulp of ripe kiwifruits was also comparable to those obtained from other plant extracts: the yields (bovine milk) obtained using latex from the plants of *Euphorbiaceae* family as a coagulant ranged from 20.73% (for *E. tirucalli)* to 21.30% (for *E. nerifolia)* [38]. Furthermore, enzymes from sunflower used to coagulate cow milk showed a curd yield of 20.78% [39]. Finally, lower yields than that obtained using the preparation from pulp of ripe kiwifruits occurred when using several vegetable coagulants from berries of *Solanum elaeagnifolium* (17.77%) [40], melon, and ginger (15.1% and 15.4%, respectively) [17]. 

The high curd yield achieved in the present study may also have been related to the use of ripe kiwifruits and to the extraction method proposed, which was based on a fast and cheap procedure that was free of any added chemicals. 

## 5. Conclusions

The extraction procedure for the kiwifruit aqueous extract proposed in the present study is a fast, cheap, -free, and ecofriendly technology to obtain an aqueous kiwifruit extract. The extract obtained from both the pulp and peel of the fruit at different ripening degrees exhibited different hydrolytic actions on κ-casein, suggesting that the actinidin concentration was influenced by the fruit ripening. Further studies on cheesemaking are ongoing to develop cheese types with desirable organoleptic and textual characteristics for industrial-scale cheese production.

## Figures and Tables

**Table 1:** Aqueous extract yields from ripe and unripe kiwifruit tissues expressed as mL of obtained juice/kg of kiwifruit and as percentage of dry matter (DM) of the extract.

Tissue	Ripe		Unripe	
	Extract (mL/kg)	DM (%)	Extract (mL/kg)	DM (%)
**Pulp**	457 ± 2.5	17.5 ± 0.3	364 ± 3.1	14.5 ± 0.6
**Peel**	15 ± 2.1	12.4 ± 1.1	11 ± 1.8	10.8 ± 0.8
**Whole fruits**	469 ± 1.5	16.8 ± 0.4	371 ± 1.1	15.7 ± 0.9

**Table 2:** Effect of pH on MCA/PA ratio at 40 °C. MCA was expressed in SU/mL and PA was expressed in U.

pH						
	5.2	5.5	6.0	6.5	7.0	7.5
**MCA/PA**	7.43 ± 0.4 ^b^	9.1 ± 0.5 ^a^	5.1 ± 0.5 ^c^	3.7 ± 0.9 ^c^	1.3 ± 0.8 ^d^	1.1 ± 0.5 ^d^

Different lowercase letters (a, b, c and d) indicate a significant difference among samples at *p* < 0.05 (ANOVA with Tukey’s post hoc test).

**Table 3:** Comparison between yield of curds obtained with chymosin, microbial coagulant, and different aliquots of aqueous extract from ripe kiwifruit.

	Ripe Aqueous Extract				Chymosin	Microbial
Inoculum (*v*/*v*)	4%	3%	2%	1.6%	0.04%	0.1%
**Yield** (%)	20.03 ± 1.84 ^a^	20.27 ± 1.16 ^a^	11.43 ± 1.96 ^b^	10.90 ± 0.79 ^b^	20.93 ± 0.90 ^a^	20.06 ± 0.70 ^a^

The values are the means of data from three replications. Data are reported as percentage value, and the standard deviation was calculated using three replications. Different lowercase letters (a and b) indicate a significant difference among samples at *p* < 0.05 (ANOVA with Tukey’s post hoc test).

## Data Availability

The data presented is contained within the article.
